# Supplementary material for: Sofosbuvir terminated RNA is more resistant to SARS-CoV-2 proofreader than RNA terminated by Remdesivir
Source: Sci Rep. 2020 Oct 6;10:16577. doi: 10.1038/s41598-020-73641-9 (PMC7538426; doi:10.1038/s41598-020-73641-9)
Supplement: Supplementary file 1 — Supplementary Information. [file 41598_2020_73641_MOESM1_ESM.pdf]

Supplementary Information

Sofosbuvir Terminated RNA is More Resistant to SARS-CoV-2 Proofreader than RNA Terminated by Remdesivir

Steffen Jockusch[1,4,#], Chuanjuan Tao[1,2,#], Xiaoxu Li[1,2], Minchen Chien[1,2], Shiv Kumar[1,2], Irina Morozova[1,2], Sergey Kalachikov[1,2], James J. Russo[1,2], Jingyue Ju[1,2,3,*]

[1]Center for Genome Technology and Biomolecular Engineering; Departments of [2]Chemical Engineering, [3]Molecular Pharmacology and Therapeutics, and [4]Chemistry, Columbia University, New York, NY 10027

[#]SJ and CT contributed equally to this work.

*To whom correspondence should be addressed. Email: dj222@columbia.edu.

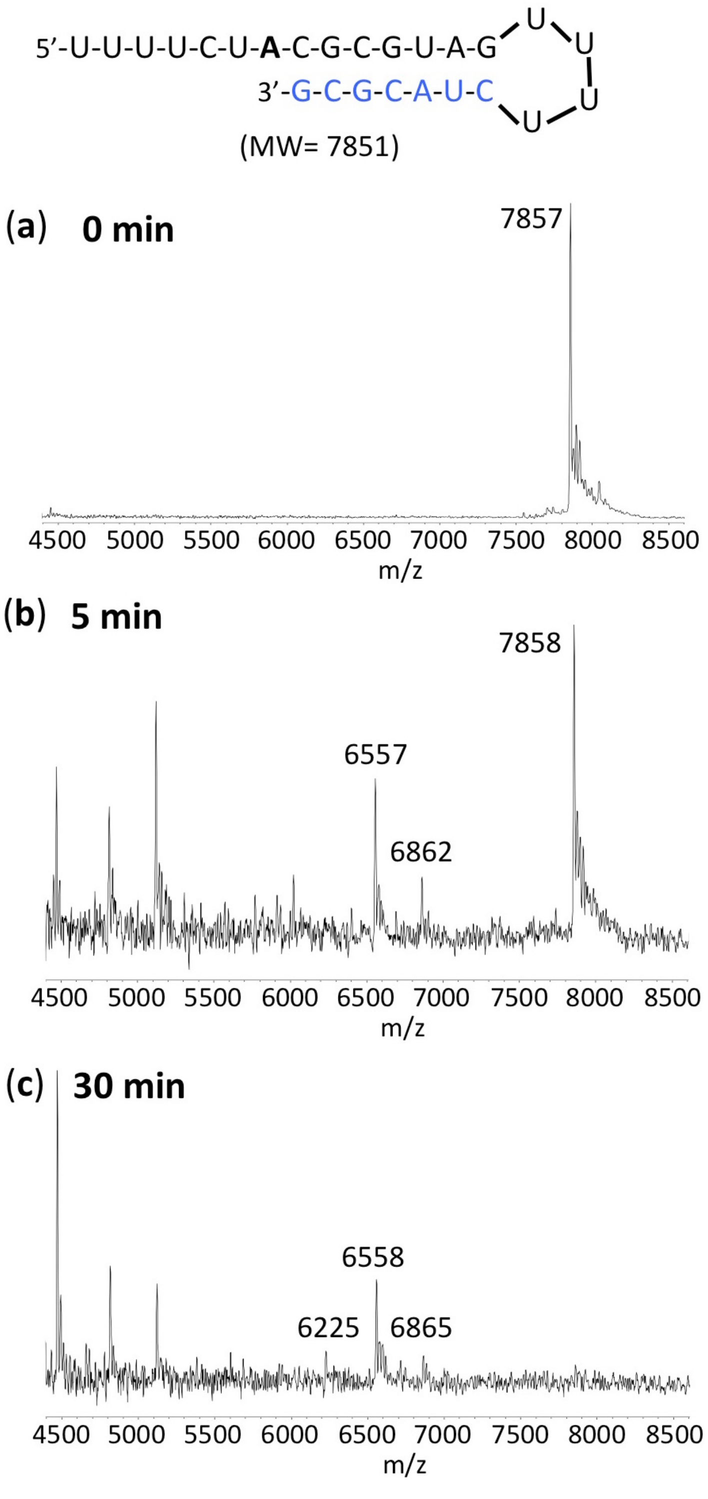

Supplementary Fig. S1. Treatment of the unextended RNA template-loop-primer (sequence shown at the top of the figure) with exonuclease as a control and analyzed by MALDI-TOF MS. The untreated RNA (0 min) is shown in (a). Exonuclease reactions were performed by incubating the RNA with the preassembled SARS-CoV-2 exonuclease complex (nsp14 and nsp10) for 5 min (b) or 30 min (c), followed by detection of reaction products by MALDI-TOF MS. The signal intensities were normalized to the highest peak. The accuracy for m/z determination is approximately ± 10 Da.
